# Quantitative imaging of individual bacterial cells: *E. coli* and *B. subtilis* via terahertz scattering-type scanning near-field optical microscopy

**DOI:** 10.1117/1.JBO.30.9.096006

**Published:** 2025-09-27

**Authors:** Haneol Lee, Youngil Moon, Donghyun Lee, Jinwoo Kim, Gyuseok Lee, Haewook Han

**Affiliations:** a Pohang University of Science and Technology (POSTECH), Department of Electrical Engineering, Pohang, Republic of Korea; b University of Arkansas, Department of Biological and Agricultural Engineering and Institute for Nanoscience and Engineering, Fayetteville, Arkansas, United States; c Korea Food Research Institute, Smart Manufacturing Research Group, Wanju, Republic of Korea

**Keywords:** terahertz scattering-type scanning near-field optical microscopy, THz imaging, near-field microscopy, single-cell, label-free imaging

## Abstract

**Significance:**

Terahertz (THz) waves have gained significant attention as an imaging technology due to their ability to provide physical and chemical information in a label-free, noninvasive, and nonionizing manner. Notably, their low energy enables nondestructive inspection of internal structures without damaging samples, making them well-suited for biomedical applications. However, the use of THz imaging has been constrained by limited spatial resolution due to the diffraction limit.

**Aim:**

This study introduces an approach using THz scattering-type scanning near-field optical microscopy, an advanced technique capable of overcoming these limitations and enabling single-cell scale measurements to image and distinguish individual bacterial cells, specifically *Escherichia coli* and *Bacillus subtilis*, representing Gram-negative and Gram-positive bacteria, respectively.

**Approach:**

We utilized tungsten vertical nanoprobes in an apertureless setup to achieve high-resolution imaging.

**Results:**

In our experiments, bacteria were measured on a hydrophilic gold substrate with a spatial resolution of 50 nm, demonstrating excellent resolution and image contrast. In addition, quantitative analysis using the line dipole image method allowed calculation of the complex refractive indices, revealing clear differences between the two bacterial species.

**Conclusions:**

This technique offers a nonlabel, noninvasive method for bacterial identification, with promising implications for advanced biomedical applications.

## 1 Introduction

Bacteria play a complex role in ecosystems, providing not only benefits but also posing threats. Beneficial bacteria are vital for nutrient cycling, aiding plant growth, and enhancing resistance to environmental stresses. For instance, nitrogen-fixing bacteria enable plants to access essential nutrients from the soil, thereby boosting growth and productivity. In addition, some beneficial bacteria help protect plants by suppressing pathogens and stimulating their immune systems.^1^^,^^2^ By contrast, pathogenic bacteria can cause significant harm to plants and animals. These harmful bacteria infect plants, leading to reduced yields and food security concerns. For example, bacterial pathogens such as Pseudomonas syringae, Xanthomonas spp., and Ralstonia solanacearum are known to cause devastating diseases in many plant species.^3^^–^^6^ In animals, they may cause a range of infections, some of which can be severe or even fatal.^7^^–^^9^ The spread of these pathogens can lead to widespread health issues among animal populations.

Understanding the structure and morphology of these pathogenic bacteria through research is crucial for numerous biomedical studies related to the diagnosis and treatment of bacterial infections. In particular, determining spectral information corresponding to the bacterial fingerprint can be used to distinguish pathogenic bacteria from harmless ones.^10^^,^^11^ This approach is significant as it ensures accurate diagnosis, which is a key factor in the success of any treatment, enabling the development of effective treatments and preventive measures. Spectral information reflects the biochemical characteristics of bacteria, enabling rapid and precise identification of specific pathogens.^12^^,^^13^ By precisely characterizing these bacterial properties, future diagnostic and therapeutic methods can be better designed, ultimately contributing to improved public health.

One common method for distinguishing pathogenic bacteria is through staining with fluorescent dyes.^14^ This technique involves applying fluorescent dyes that bind specifically to certain components of bacterial cells, making them visible under a fluorescence microscope. However, this method is destructive and irreversible to the samples as the staining process often requires fixing the bacteria, which kills them and makes it impossible to perform any subsequent live-cell analyses. Furthermore, methods such as staining often presuppose a sufficient quantity of cells, typically achieved through culturing. By contrast, terahertz scattering-type scanning near-field optical microscopy (THz s-SNOM) offers the advantage of a simplified sample preparation process. Ideally, as analysis is possible with even a single bacterium, the sample amplification step required for culturing can be eliminated.^15^ This is particularly problematic for samples where bacterial culture conditions are unknown. Even if some bacterial samples are obtained, fluorescent staining is ineffective when multiple bacterial species are present in a mixed sample. This is because the dye cannot differentiate between different species, leading to ambiguous or misleading results. Consequently, there is a growing demand for methods that enable individual bacterial analysis without destructive staining or extensive culture.^16^

Conventional microscopy techniques, such as bright-field, phase-contrast, and electron microscopy, are common tools used to analyze the structural and biochemical characteristics of various bacteria. Bright-field microscopy provides basic morphological information, whereas phase-contrast microscopy enhances the contrast of transparent specimens without the need for staining. Conversely, electron microscopy offers high-resolution images of bacterial ultrastructure but requires operation under high-vacuum conditions and often involves destructive preparation steps such as metal coating or ultra-thin sectioning, which can alter the intrinsic properties of the sample.^17^^,^^18^

Although widely used, conventional optical microscopy techniques are fundamentally constrained by the diffraction limit, which restricts the resolution needed for single-cell scale analysis.^19^ The spatial resolution of laser-based optical microscopy is limited to ∼200 to 250 nm, about half the wavelength of light, which is insufficient for resolving subcellular structures in bacterial cells, typically 1 to 2  μm, where effective imaging requires a much finer resolution of 50 to 100 nm.^20^ Recently, Lucidi et al.^21^ demonstrated stimulated emission depletion nanoscopy of KK114-stained pathogenic bacteria, visualizing cell envelope structures at 30 to 50 nm resolution. However, these super-resolution optical techniques depend on fluorescence labeling and require shorter-wavelength light, the higher photon energy of which can damage bacterial cells. High-energy photons may induce phototoxicity and photobleaching, compromising sample integrity and resulting accuracy.^22^

These limitations can be overcome by using terahertz (THz) waves to analyze bacteria. THz waves refer to electromagnetic waves within the frequency range of 0.1 to 10 THz, corresponding to wavelengths from 3 mm to 30  μm and energy from 0.4 to 40 meV in the electromagnetic spectrum. THz waves have low photon energy well below the threshold needed for ionization or bond dissociation, rendering THz radiation fundamentally nondestructive for biological samples, in contrast to higher energy radiation such as X-rays or ultraviolet light.^23^^–^^25^ Although THz waves are strongly absorbed by polar molecules such as water owing to resonant interactions with vibrational and rotational modes, they can readily penetrate many nonpolar materials (such as plastics, ceramics, and dry biological tissues) that are opaque at other wavelengths.^26^^,^^27^ However, they also interact with unique low-frequency collective modes in biomolecules, such as protein folding and hydration dynamics. This sensitivity to biomolecules and their aqueous environment has led to advancements in THz imaging technology.^28^^–^^31^

Although infrared (IR)-band s-SNOM excels at surface chemical contrast via mid-IR vibrational modes, THz s-SNOM offers distinct advantages: its much lower photon energy enables label-free, nondestructive imaging.^26^ Strong THz absorption by water allows quantitative mapping of hydration states,^26^ and its long-wavelength near-field interaction probes subsurface dielectric contrasts inaccessible to IR.^32^ These capabilities uniquely position THz s-SNOM for comprehensive, nanoscale biochemical heterogeneity imaging within bacterial cells.

THz imaging offers significant advantages by providing physical and chemical information about samples in a label-free, noninvasive, and nonionizing manner.^25^^,^^33^ This is particularly important in biological research and medical diagnostics as THz waves can analyze internal tissues without causing harm. For example, THz imaging can be used to noninvasively diagnose diseases such as skin cancer in the early stages.^34^ Numerous studies have recently reported on imaging biological samples using THz waves.^35^^–^^37^ Studies using THz imaging, for instance, have provided insights into the structure and dynamics of biomolecular systems, such as protein folding and hydration dynamics.^26^^,^^27^ However, leveraging this unique THz contrast to study subcellular structures necessitates overcoming the significant challenge posed by long wavelengths, which inherently limit spatial resolution.^38^

THz s-SNOM has emerged as a cutting-edge technique for addressing the resolution limitations of conventional THz imaging. This powerful technology integrates THz optics, employing components utilized in THz time-domain spectroscopy (THz-TDS), with the precise nanoscale positioning and topographic feedback of an atomic force microscope (AFM).^39^ THz s-SNOM operates by illuminating a sharp metallic AFM tip with focused THz radiation; the tip confines the THz interaction to a nanoscale volume directly beneath it and scatters the near-field signal containing local sample information. By detecting this tip-scattered near-field signal, THz s-SNOM bypasses the diffraction limit, achieving spatial resolutions typically in the range of tens of nanometers, orders of magnitude better than conventional far-field THz microscopy. Notably, in the near-field system, the spatial resolution is determined by the sharpness of the probe tip, specifically its effective radius, rather than by the wavelength of the incident THz radiation.^38^ The THz near-field signal around the probe tip provides contrast based on the local dielectric function or conductivity of the sample at THz frequencies, offering label-free mapping of material properties and chemical composition with nanoscale detail at the THz frequency range.^32^ This capability to perform high-resolution THz imaging and spectroscopy unlocks new possibilities for investigating the intricate structures and nanoscale phenomena within diverse biological and material systems.^40^^–^^43^

In this study, we demonstrate the application of THz s-SNOM for quantitative, label-free imaging and differentiation of bacterial cells. In particular, we employed an apertureless THz s-SNOM setup with a tungsten vertical nanoprobe to measure the near-field signals from individual *Escherichia coli* (*E. coli*) (Gram-negative) and *Bacillus subtilis* (*B. subtilis*) (Gram-positive) bacteria prepared on a hydrophilic gold substrate. Experimental results were quantitatively analyzed by comparing measured signal amplitudes, supported by theoretical calculations using the line dipole image method (LDIM).^44^^–^^47^ Our findings reveal distinct THz signal contrasts between the two bacterial species, achieving nanoscale spatial resolution (∼50  nm) and demonstrating the potential of THz s-SNOM for noninvasive bacterial identification.

## 2 Methods

### 2.1 Sample Preparation

**Fig. 1:**
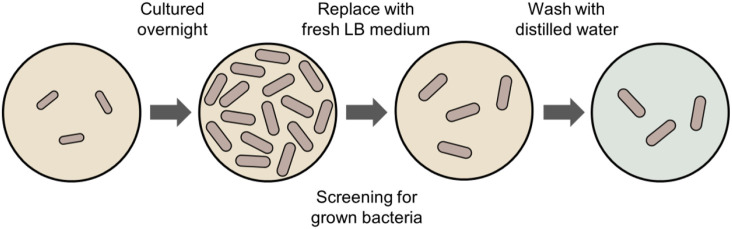
Schematic illustration of the bacterial sample preparation process.

We prepared the representative Gram-negative bacteria *E. coli* (W3110 strain) and Gram-positive bacteria *B. subtilis* using the process shown in Fig. 1. For the bacterial culture, each strain was first plated on Luria–Bertani (LB) agar plates (10  g/L tryptone, 10  g/L NaCl, 5  g/L yeast extract, 15  g/L agar, pH 7.0). A single colony was selected, inoculated into a test tube containing LB broth (10  g/L tryptone, 10  g/L NaCl, 5  g/L yeast extract, pH 7.0), and cultured overnight at 37°C with shaking. A portion of the overnight culture was then reinoculated into fresh LB broth and further cultured for 5 to 6 h at the same temperature. During this period, bacterial growth was monitored by measuring the optical density at 600 nm using a spectrometer (UV-1700, Shimadzu, Japan) to ensure cells reached the desired growth phase, such as the late-logarithmic phase.

After cultivation, the bacterial cells underwent a washing process before analysis. The cell culture was centrifuged (at 5000 rpm for 10 min at room temperature) to obtain a cell pellet. The supernatant was subsequently removed, and the cell pellet was resuspended in distilled (DI) water. This washing step, involving centrifugation and resuspension, was repeated 3 times. Finally, the washed cells were diluted in DI water to prepare a cell suspension with a concentration of ∼2000  cells/μL.

A thin gold (Au) film was used as the substrate for measuring the THz near-field signal of the bacterial samples. The initially hydrophobic surface of the gold film was treated using an inductively coupled plasma dry etching system to render the surface hydrophilic. This modification aimed to ensure uniform spreading of the aqueous bacterial suspension on the substrate surface. A small volume (1  μL) of the final diluted bacterial suspension was drop-cast onto the prepared hydrophilic gold substrate and subsequently air-dried under ambient conditions to fabricate the sample for THz s-SNOM measurements.

In our THz s-SNOM setup, the near-field signal is generated from THz radiation that passes through the sample and reflects off the substrate. Therefore, a substrate with high reflectivity in the THz range is essential. Gold is well known for its excellent THz reflectivity (≈98%); however, untreated gold surfaces are hydrophobic and unsuitable for bacterial adhesion. To address this, we functionalized the gold substrate to render it hydrophilic, enabling stable bacterial fixation. Standard substrates such as glass (≈30% to 40% reflectivity), silicon (≈60% to 70%), or quartz exhibit significantly lower THz reflectivity and cannot replace gold under our imaging conditions. Although gold is optimal for the current setup, future studies may investigate biocompatible, high-reflectivity substrates such as indium tin oxide (ITO) or nanostructured gold coatings.

We initially cultured ∼2000 cells per species and selected 10 morphologically similar bacterial cells using optical microscopy. Due to the >40  h imaging time per cell and occasional signal instability or probe tip damage, complete datasets were obtained for only six independent imaging replicates per species. All quantitative results are reported as descriptive statistics (mean ± SD for N=6). No inferential statistics were applied due to the limited sample size.

All THz s-SNOM measurements were performed in a dry air environment after complete removal of atmospheric moisture as THz waves are strongly absorbed by water vapor. Imaging was conducted using a raster scanning method, measuring the THz signal pixel by pixel and moving the probe between pixels, a process that requires tens of hours per cell. As a result, bacterial cells were fixed and dried before imaging, and real-time live-cell imaging was not performed.

### 2.2 Experimental Setup

**Fig. 2:**
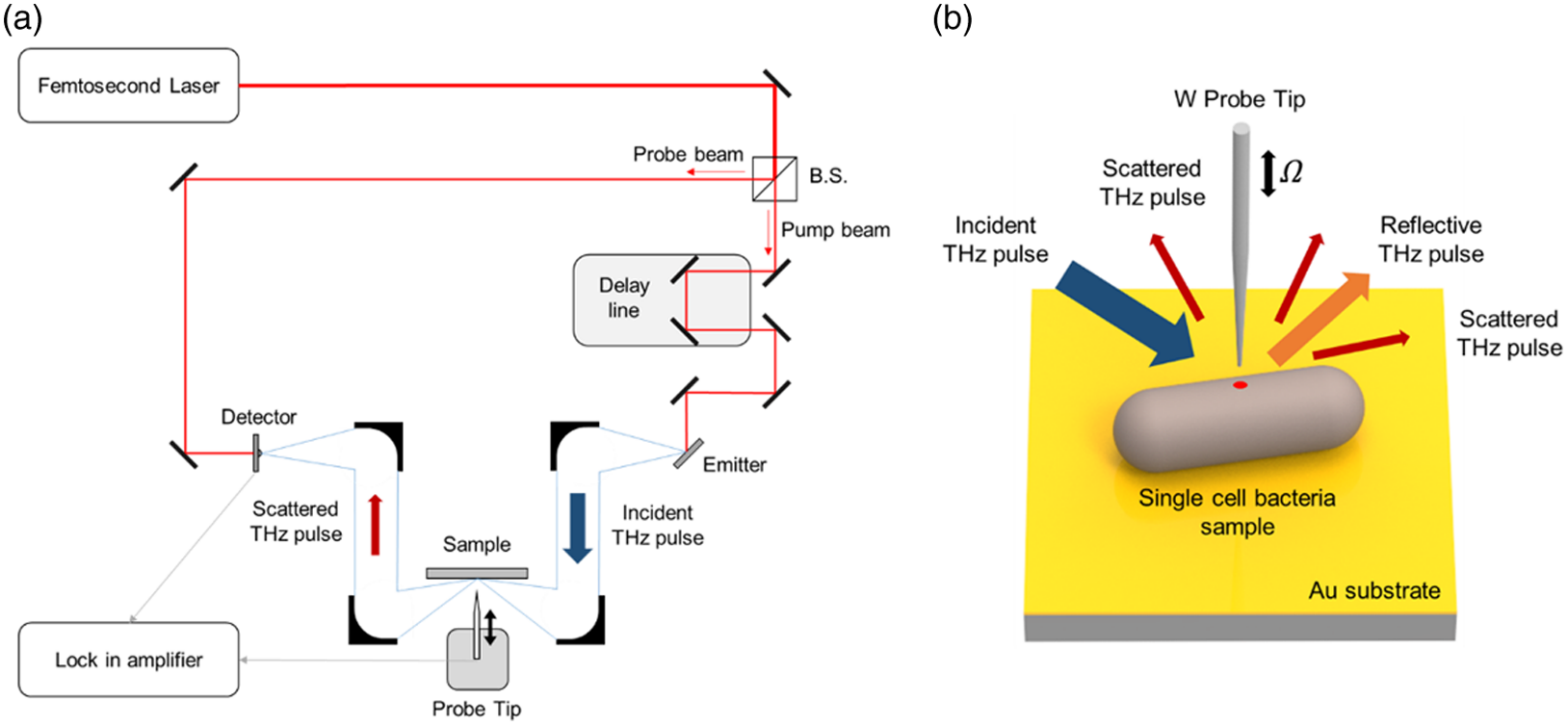
Schematic of the THz s-SNOM experimental setup. (a) Configuration of the THz-TDS-based s-SNOM system integrated with an AFM. (b) Illustration of the THz near-field interaction involving the oscillating W probe tip and a bacterial cell on an Au substrate. THz s-SNOM, THz scattering-type scanning near-field optical microscopy; THz-TDS, THz time-domain spectroscopy; AFM, atomic force microscope; W, tungsten; and Au: gold.

The THz s-SNOM system (Fig. 2) used to image bacterial cells integrated a custom-built tapping-mode AFM with a standard THz-TDS setup. Ti:sapphire ultrafast laser (MaiTai, Spectra-Physics, Milpitas, California, United States) was used as the optical source for generating and coherently detecting THz pulses within the THz-TDS system. The THz pulses were generated by an indium arsenide wafer emitter with focused femtosecond laser pulses. The generated THz beam was subsequently collimated and focused onto the AFM probe tip region using a series of gold-coated off-axis parabolic mirrors (OAPMs). The optical path was aligned such that the incident THz beam was p-polarized and impinged on the sample at an angle of ∼60  deg with respect to the sample surface normal.

The AFM employed a sharp conductive probe tip fabricated by electrochemical etching of a tungsten (W) wire, starting from an initial diameter of 50  μm. The tip apex was etched down to a radius of ∼50  nm to optimize the spatial resolution for imaging individual bacterial cells in this study. The effective apex radius of the probe was measured directly by SEM after completion of all imaging experiments. This tip was securely attached to one prong of a quartz tuning fork, which acted as the oscillatory element for tapping-mode operation. The tuning fork was mechanically dithered precisely at its resonance frequency of 28 kHz in this setup. The oscillation signal was utilized within a feedback loop to maintain a constant tip–sample distance with nanometer precision. The sample, prepared as described in Sec. 2.1, was mounted on a precision three-axis nano-positioning stage that performed raster scanning in the x−y plane beneath the stationary probe tip to acquire surface data.

The THz radiation scattered from the near-field interaction zone between the tip and the sample was efficiently collected by another OAPM and directed toward the detector. Coherent detection of the scattered THz electric field was accomplished using a photoconductive antenna (PCA) fabricated on a low-temperature–grown GaAs (LTG-GaAs) substrate, which was gated by time-delayed pulses derived from the same femtosecond laser source. The photocurrent output from the PCA detector was fed into a lock-in amplifier for demodulation at the first harmonic (n=1, 1  Ω) of the tapping frequency of the tip (Ω=28  kHz). This was done to maximize the signal-to-noise ratio for the near-field signal. In our previous work,^47^ we demonstrated that in a THz-TDS–based s-SNOM configuration, first-harmonic detection yields a substantially longer probe–sample interaction distance (e.g., $E_{1}=38$, $E_{2}=15$, and $E_{3}=13\;\mathrm{nm}$ on Au substrates) and offers comparable lateral resolution (∼90  nm), with negligible background artifacts. These findings suggest that higher harmonic or pseudoheterodyne detection schemes are unnecessary for THz s-SNOM. Accordingly, we employ first-harmonic demodulation in the present study. Time-domain waveforms at each scan point were acquired by scanning an optical delay line incorporated in the THz-TDS setup. Frequency-domain spectra were subsequently obtained by applying a fast Fourier transform to the recorded time-domain signals.

The entire THz s-SNOM system was enclosed within a sealed chamber purged with dry air to ensure reliable measurements by eliminating the strong absorption of THz waves by ambient water vapor. Throughout all experiments, the internal environment within the chamber was maintained at a low humidity level, corresponding to a dew point below −55°C.

### 2.3 Calculation of Complex Refractive Index

The quantitative determination of the frequency-dependent complex refractive index, $\tilde{n}(\omega )=n(\omega )+i\kappa (\omega )$, or equivalently, the complex dielectric function, $\varepsilon (\omega )=\varepsilon^{\prime }(\omega )+i\varepsilon^{\prime \prime }(\omega )$, of the bacterial cells is based on a rigorous analysis of the complex near-field scattering signal acquired by the THz s-SNOM system. This analysis utilizes the signal components corresponding to the first harmonic (1  Ω) of the tapping frequency (Ω) of the tip of the probe, obtained via the demodulation process detailed in Sec. 2.2. The first harmonic is typically employed as it generally offers the highest signal-to-noise ratio, which is advantageous in accurately extracting spectral features.^48^ The resulting complex near-field scattering signal is characterized by its measured spectral amplitude $S_{\left(1\right)}(\omega )$ and phase $\phi_{\left(1\right)}(\omega )$ at each angular frequency ω and spatial point

$$E_{\mathrm{sc}(1)}(\omega )=S_{\left(1\right)}(\omega )\cdot e^{i\phi_{\left(1\right)}(\omega )}.$$ (1)

These measured amplitude and phase components serve as direct inputs for the theoretical modeling and calculations presented subsequently to determine the intrinsic optical constants of the sample.

A theoretical model describing the electromagnetic interaction between the AFM probe tip and the sample surface is necessary to quantitatively analyze the complex near-field scattering signal, $E_{\mathrm{sc}(1)}(\omega )$, and extract the optical constants of the sample. Given that the effective radius of the probe apex (a) is significantly smaller than the incident THz wavelength (a≪λ), the interaction can be accurately described within the quasistatic approximation.^44^ For analytical modeling within this regime, the sharp probe apex is commonly approximated as a polarizable sphere with an effective radius a and complex permittivity $\epsilon_{p}$. The point dipole image method (PDIM) represents a simplified approach based on this sphere model.^49^ In PDIM, the initial dipole moment induced in the probe sphere, $d_{p}^{\mathrm{PD}}$, arises solely from the incident electric field, $E_{\text{in}}$

$$d_{p}^{\mathrm{PD}}=\alpha E_{\text{in}},$$ (2)

where $d_{p}^{\mathrm{PD}}$ is the initial probe dipole moment in the PDIM, $\alpha =4\pi \epsilon_{0}a^{3}(\epsilon_{p}-1)/(\epsilon_{p}+2)$ is the Clausius–Mossotti polarizability of the probe sphere, $E_{\text{in}}$ is the incident electric field vector, and $\epsilon_{p}$ is probe permittivity. Although PDIM offers simplicity, the LDIM provides a more rigorous description by incorporating self-consistent image theory to better satisfy boundary conditions.^44^^,^^45^ In the LDIM model, the initial probe dipole moment, denoted as $d_{p}^{\mathrm{LD}}$ (representing the first iteration step), considers the incident field and the field reflected from the sample surface, expressed as

$$d_{p(1)}^{\mathrm{LD}}=\alpha (1+r)E_{\text{in}},$$ (3)

where $d_{p(1)}^{\mathrm{LD}}$ is the initial probe dipole moment in the LDIM (first iteration) and r is the reflection coefficient of the sample surface for the incident field. The inclusion of the reflected field contribution (r) in the initial probe dipole calculation is a key distinction of the LDIM, leading to a more accurate representation of the near-field interaction compared with the PDIM, particularly for quantitative studies. Subsequent steps in both models involve calculating further interactions with image dipoles in the substrate. However, this difference in the initial probe polarization highlights the improved physical basis of the LDIM.

The LDIM computation proceeds iteratively to self-consistently determine the near-field interaction, moving beyond the single-point dipole approximation of the PDIM. Starting from the initial probe dipole $d_{p(1)}^{\mathrm{LD}}$ (which includes substrate reflection), an image dipole is induced in the sample. This image dipole, in turn, induces a point dipole and a continuous line dipole density within the probe sphere in the subsequent iteration, calculated using the substrate–probe image generation dyadic (γ). This induced probe density then creates an image density within the sample via the probe–substrate reflection image generation dyadic (β_T), and this process repeats recursively. The line dipole densities in the probe, $p_{p(n)}^{\mathrm{LD}}(z,h)$, and the sample, $p_{s(n)}^{\mathrm{LD}}(z,h)$, for the n’th iteration (n≥2) are calculated as^45^

$$p_{p(n)}^{\mathrm{LD}}(z,h)=\int_{-h}^{a-h}dz^{\prime }\gamma (z,h,h-z^{\prime })\cdot p_{s(n-1)}^{\mathrm{LD}}(z^{\prime },h)$$ (4)

$$p_{s(n)}^{\mathrm{LD}}(z,h)=\beta_{T}\cdot p_{p(n-1)}^{\mathrm{LD}}(-z,h),$$ (5)

where $p_{p(n)}^{\mathrm{LD}}(z,h)$ and $p_{s(n)}^{\mathrm{LD}}(z,h)$ are the probe and sample line dipole densities at iteration n and position z, respectively, h=a+g is the distance from the sphere center to the substrate, γ(z,h,L) is the substrate–probe image generation dyadic, relating a dipole density in the substrate to the induced density in the probe, and $\beta_{T}$ is the probe–substrate reflection image generation dyadic, related to the reflection coefficient β of the sample. The total induced dipole moment in the tip–substrate system, $d^{\mathrm{LD}}(h)$, which accurately captures the complex, nonlinear dependence on tip–sample distance h (and material properties), is obtained by integrating and summing these line dipole densities across all iterations as follows:

$$d^{\mathrm{LD}}(h)=\overset{\infty }{\underset{n=1}{\sum }}[\int_{h-a}^{h}dz∼p_{p(n)}^{\mathrm{LD}}(z,h)+\int_{-h}^{a-h}dz∼p_{s(n)}^{\mathrm{LD}}(z,h)].$$ (6)

Notably, the experimentally measured complex near-field signal at the first harmonic, $E_{\mathrm{sc}(1)}(\omega )$, is directly proportional to the first harmonic component of this rigorously calculated total dipole moment, $d^{\mathrm{LD}}(h,\omega )$. This provides the theoretical basis for relating the measured signal to the intrinsic properties of the sample in the subsequent analysis steps.

The measured complex near-field signals require normalization to utilize the relationship derived from the LDIM and extract intrinsic material properties while accounting for experimental factors such as tip geometry and system response. The complex signal acquired from the bacterial sample [amplitude $S_{\text{sample},1}(\omega )$, phase $\phi_{\text{sample},1}(\omega )$] is divided by the signal obtained from a suitable reference material (e.g., gold or the bare substrate), amplitude $S_{\mathrm{ref},1}(\omega )$, and phase $e^{i\phi_{\mathrm{ref},1}(\omega )}$ measured under identical conditions. This procedure yields the normalized complex scattering signal, $\sigma_{\text{norm},1}(\omega )$, as follows:^46^^,^^48^

$$\sigma_{\text{norm},1}(\omega )=\frac{S_{\text{sample},1}(\omega )\cdot e^{i\phi_{\text{sample},1}(\omega )}}{S_{\mathrm{ref},1}(\omega )\cdot e^{i\phi_{\mathrm{ref},1}(\omega )}}=(\frac{S_{\text{sample},1}(\omega )}{S_{\mathrm{ref},1}(\omega )})\cdot e^{i[\phi_{\text{sample},1}(\omega )-\phi_{\mathrm{ref},1}(\omega )]}.$$ (7)

This normalized signal $\sigma_{\text{norm},1}(\omega )$, effectively representing the response of the sample relative to the reference, is then interpreted within the LDIM model. A widely adopted approximation for quantitative analysis relates this normalized signal to the effective reflection coefficients, β=(ϵ−1)/(ϵ+1), of the sample and reference materials as follows:

$$\sigma_{\text{norm},1}(\omega )\approx \frac{\beta_{\text{sample}}(\omega )}{\beta_{\mathrm{ref}}(\omega )}=\frac{\left(\epsilon_{\text{sample}}(\omega )-1)/(\epsilon_{\text{sample}}(\omega )+1\right)}{\left(\epsilon_{\mathrm{ref}}(\omega )-1)/(\epsilon_{\mathrm{ref}}(\omega )+1\right)},$$ (8)

where $\beta_{\text{sample}}(\omega )$ and $\beta_{\mathrm{ref}}(\omega )$ are the effective reflection coefficients for the sample and reference, $\epsilon_{\text{sample}}(\omega )$ is the complex dielectric function of the bacterial sample (unknown), and $\epsilon_{\mathrm{ref}}(\omega )$ is the complex dielectric function of the reference material (known). By employing the experimentally determined complex value of $\sigma_{\text{norm},1}(\omega )$ and the known complex dielectric function $\epsilon_{\mathrm{ref}}(\omega )$, the unknown complex dielectric function of the bacterial sample, $\epsilon_{\text{sample}}(\omega )$, is calculated by numerically inverting this equation at each frequency ω. Finally, the complex refractive index of the sample, ${\overset{˜}{n}}_{\text{sample}}(\omega )$, is obtained from the calculated $\epsilon_{\text{sample}}(\omega )$ via the following standard relation:

$${\overset{˜}{n}}_{\text{sample}}(\omega )=\sqrt{\epsilon_{\text{sample}}(\omega )},$$ (9)

where ${\overset{˜}{n}}_{\text{sample}}(\omega )$ is the complex refractive index of the sample and $\epsilon_{\text{sample}}(\omega )$ is the calculated complex dielectric function of the sample. This comprehensive analysis protocol, integrating LDIM-based theoretical modeling with experimental signal normalization, enables the quantitative extraction of the frequency-dependent intrinsic optical constants for bacterial cells.

Although the LDIM equations [Eqs. (2)–(6)] have been reported previously, we apply them pixel by pixel within our THz-TDS–based s-SNOM setup to quantitatively reconstruct local dielectric contrasts. This per-pixel implementation is essential for correlating the experimental amplitude maps with theoretical dielectric profiles.

## 3 Results and Discussion

**Fig. 3:**
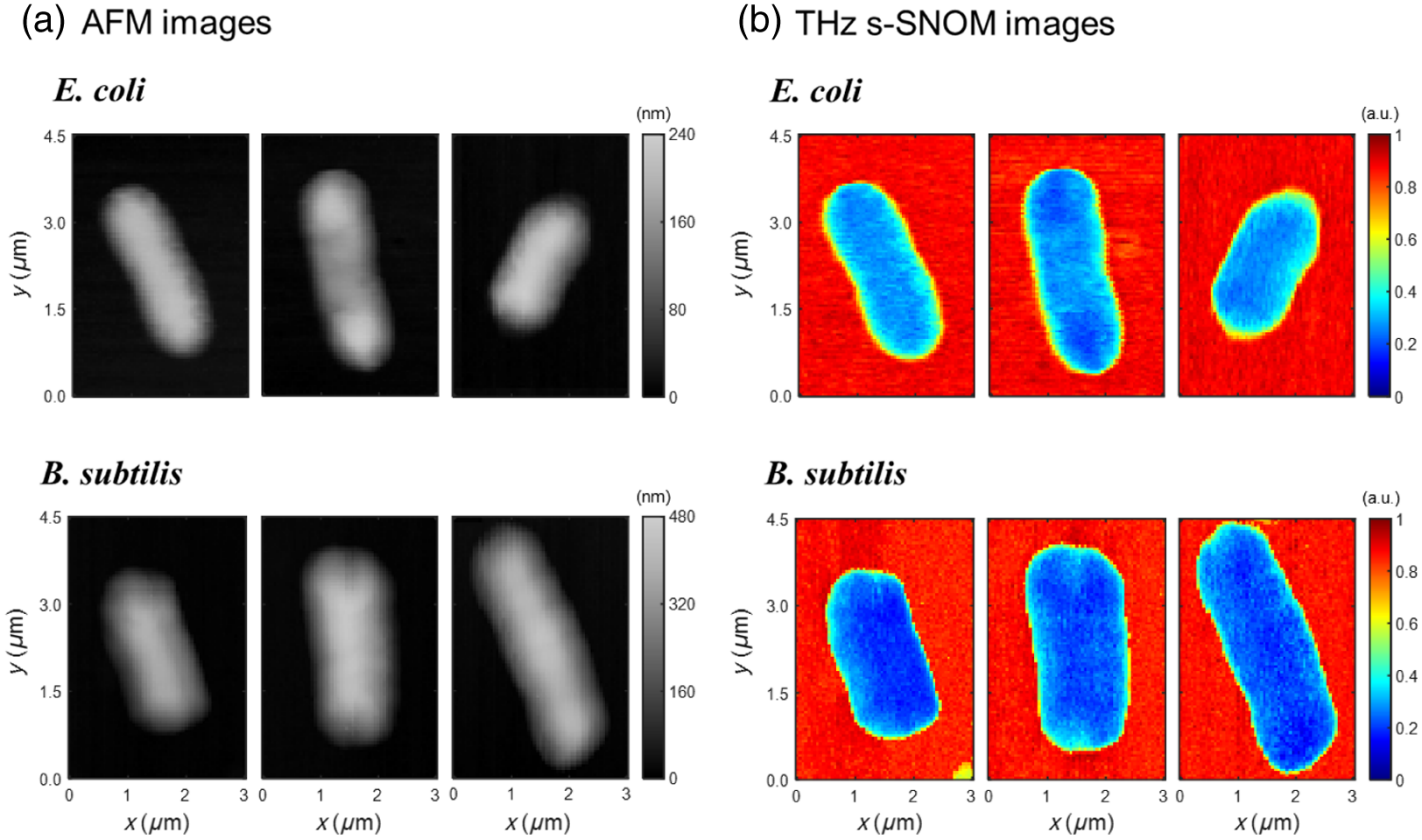
THz s-SNOM imaging of bacterial cells. (a) AFM topography maps of *E. coli* and *B. subtilis* on an Au substrate. (b) Corresponding THz s-SNOM images acquired simultaneously, where the pixel values represent the peak intensity of the scattered THz pulse measured in the time domain. These images were obtained by raster scanning using a W probe with an effective apex radius of ∼50  nm. THz s-SNOM, THz scattering-type scanning near-field optical microscopy; AFM: atomic force microscope; W, tungsten; and Au, gold.

The high-resolution imaging of individual bacterial cells with a spatial resolution of ∼50  nm was performed using the THz s-SNOM system detailed in Sec. 2. Figure 3(a) shows the AFM topography images acquired for the specific *E. coli* and *B. subtilis* cells prepared on a hydrophilic Au substrate. Both species exhibit similar rod-like shapes and dimensions, consistent with typical values for these species in the relevant growth phase, indicating successful sample preparation.^50^^,^^51^ Although they exhibit similar morphologies, *E. coli* and *B. subtilis* possess fundamentally distinct cell wall structures, resulting in Gram-negative and Gram-positive bacteria classifications, respectively. The THz images in Fig. 3(b) were constructed by mapping the peak intensity of the scattered THz pulse measured in the time domain. Indeed, Fig. 3(b) reveals a noticeable difference in the THz signal intensity between the two species; *E. coli* generally shows lower overall signal intensity than *B. subtilis* and exhibits a pattern in which lower signal intensity is distributed over the region corresponding to the cell envelope. Imaging with nanoscale resolution in the THz band enables detailed analysis and understanding of cellular structure at the single-cell level.

**Fig. 4:**
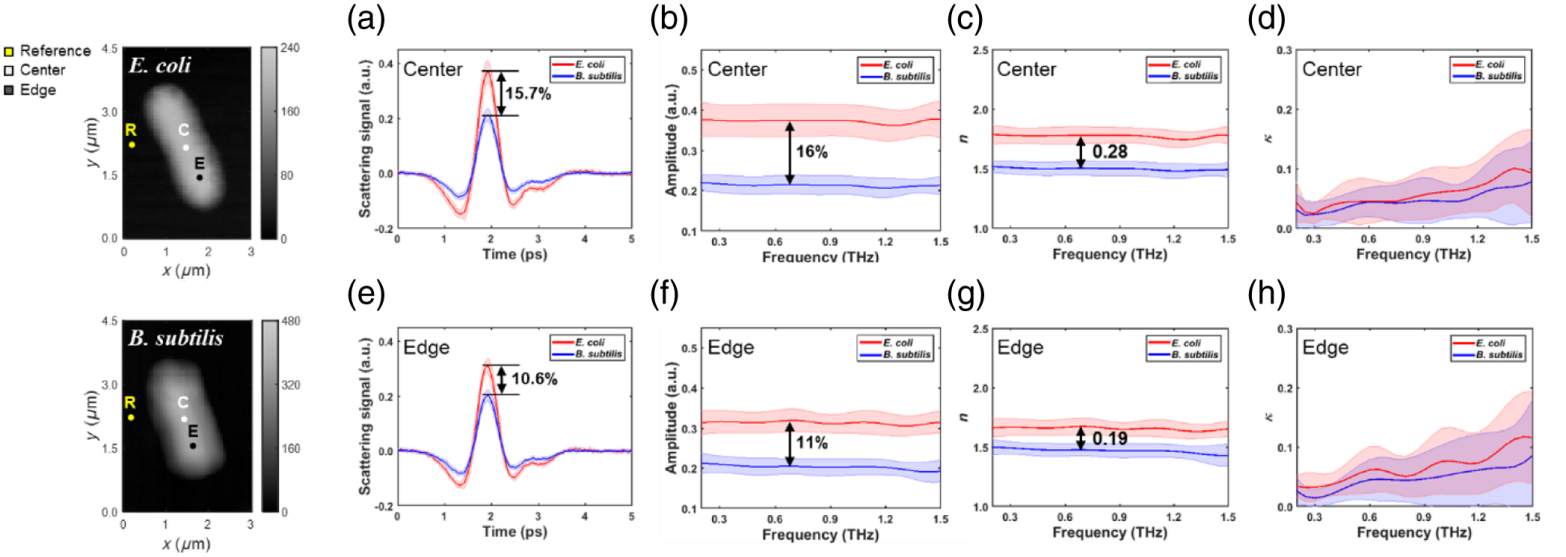
Comparative THz near-field analysis of *E. coli* (red) and *B. subtilis* (blue) at (a)–(d) cell center and (e)–(h) edge locations, indicated on AFM images (left). (a) and (e) Time-domain scattering waveforms. (b) and (f) Frequency-domain amplitude spectra. (c) and (g) Refractive index (n). (d) and (h) Extinction coefficient (κ). Each curve represents the average of measurements from six independent bacteria (N=6 biological replicates), with the solid line indicating the mean and the shaded area representing the standard deviation. For each individual bacterium, the signal at a given point was obtained by averaging 10 technical replicates.

The near-field signals were analyzed at specific locations on the cells, namely, the center and edge regions, to quantitatively differentiate the two species based on their THz response, as indicated in the AFM images (Fig. 4, left insets). The higher THz signal amplitude at the cell center reflects a larger tip–sample interaction volume that encompasses the full cell thickness and its internal hydration state. By contrast, the lower signal at the cell edge primarily arises from the thin peripheral layer and the substrate interface. This spatial variation is attributed to differences in cell thickness and water absorption.^52^^,^^53^ Comparing the time-domain scattering waveforms [Figs. 4(a) and 4(e)], the peak signal amplitude at the cell center shows a difference of ∼15.7% between *E. coli* (lower) and *B. subtilis* (higher). A similar trend, albeit with a reduced difference of ∼10.6%, is observed at the cell edge. This amplitude contrast persists across the measured spectrum, as shown in the frequency-domain amplitude plots [Figs. 4(b) and 4(f)]. The average amplitude difference between the two species is ∼16% at the center and 11% at the edge across the 0.3 to 1.5 THz range. These direct measurements of scattered signal amplitude provide a clear basis for distinguishing the bacteria. We attribute the observed species-dependent differences in THz signal amplitude to variations in cell envelope structure and hydration. *B. subtilis* (Gram-positive) has a thick peptidoglycan layer and higher water content, resulting in higher effective permittivity at THz frequencies. By contrast, *E. coli* (Gram-negative) possesses a thinner peptidoglycan layer and a complex outer membrane, leading to lower permittivity and reduced signal amplitude. Similar trends have been reported in bulk bacterial pellets using THz-TDS, where refractive index correlates with water content,^54^ and in single-cell THz near-field imaging, which reveals edge-to-center signal variation driven by cell thickness and hydration distribution.^53^ Furthermore, the complex refractive indices extracted using the LDIM model (described in Sec. 2.3) also reflect this difference. The calculated refractive index [n, Figs. 4(c) and 4(g)] for E. coli is consistently lower than that for B. subtilis, with an average difference of ∼0.28 at the center and 0.19 at the edge. Although the small number of biological replicates (N=6) precludes formal inferential statistical analysis, the mean refractive index values for each species are clearly separated and fall outside the standard deviation range of the other, suggesting a robust and reproducible contrast. The calculated extinction coefficients [κ, Fig. 4(d) and 4(h)] remain low for both species. This is attributed to the removal of bulk water, which is the primary source of absorption in this THz frequency range.

Full-area n/κ mapping using THz-TDS requires delay-line scans of 3 to 5  min/pixel. As a result, imaging a single bacterium at 100×100  pixels would require over 500 h of continuous measurement. Over such an extended acquisition period, any probe tip damage or signal instability could invalidate the entire experiment, making full-area mapping impractical under current conditions. Therefore, we performed n/κ profiling along a representative line across each bacterium to quantitatively distinguish the two species. Full-area n/κ mapping will be explored in future work to overcome these technical limitations.

**Fig. 5:**
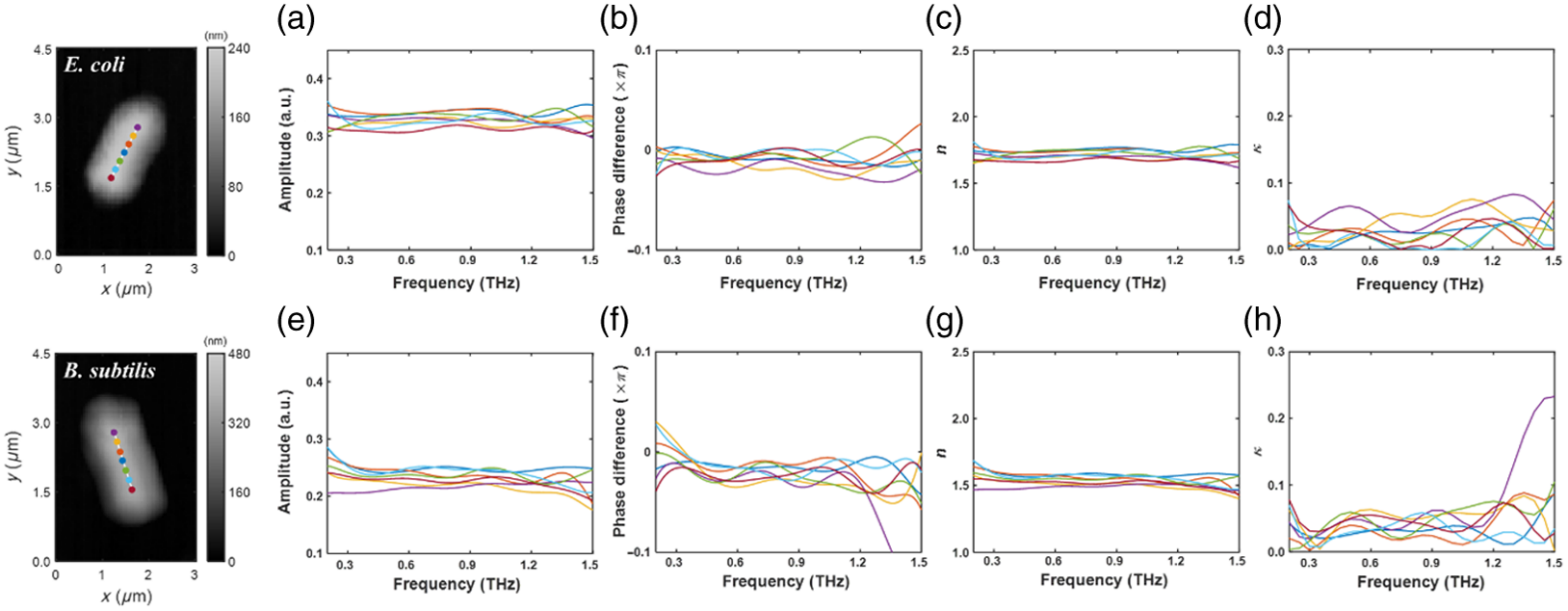
Spectroscopic analysis at multiple local points (indicated by colored dots on AFM images, left) on [top row, (a)–(d)] *E. coli* and [bottom row, (e)–(h)] *B. subtilis*. (a) and (e) Near-field amplitude spectra. (b) and (f) Near-field phase difference spectra (relative to the substrate). (c) and (g) Calculated refractive index (n). (d) and (h) Calculated extinction coefficient (κ). Each colored curve corresponds to a different spatial measurement point on a single bacterium (biological replicate, N=1). The signal at each point was obtained by averaging 10 technical replicates.

The spectroscopic data acquired from multiple distinct points along the central axis of representative cells (Fig. 5) further highlight the intrinsic THz properties and their local variations. The near-field amplitude spectra [Figs. 5(a) and 5(e)] and phase difference spectra [relative to the substrate, Figs. 5(b) and 5(f)] exhibit some variability between different measurement points on the same cell type, potentially reflecting local inhomogeneities in density or composition. However, the overall trends and average levels remain distinct between the two species. Similarly, the calculated refractive index and extinction coefficients [Figs. 5(c) and 5(g)] and [Figs. 5(d) and 5(h), respectively] show point-to-point fluctuations but maintain a clear separation in the average refractive index values between *E. coli* (lower n) and *B. subtilis* (higher n). This consistency across multiple locations strengthens the conclusion that the observed THz contrast is a reliable indicator of the bacterial species.

The observed and calculated differences in the THz optical properties between *E. coli* and *B. subtilis* can be attributed to their fundamentally different cell envelope architectures. The thick (∼20 to 80 nm) peptidoglycan layer characteristic of Gram-positive *B. subtilis* likely contributes to a higher overall effective permittivity compared with the Gram-negative *E. coli*, which possesses a much thinner (∼2 to 7 nm) peptidoglycan layer. Although the samples are dried, composition, density, and residual bound water differences associated with these distinct cell wall structures are the most probable cause for the differing dielectric responses observed at THz frequencies. This study demonstrates that by leveraging these intrinsic material differences, THz s-SNOM can effectively function as a nanoscale, label-free probe for bacterial differentiation. The achieved resolution allows measurements well below the scale of individual cells, opening possibilities for probing structure–function relationships. Although interpreting absolute dielectric values requires careful calibration and consideration of model limitations (e.g., tip shape approximation), the relative contrast provides a robust method for distinguishing these bacterial types. Although this study serves as a proof-of-concept demonstrating that bacterial species can be distinguished by their THz near-field signals, caution should be exercised when generalizing these results to other species. Therefore, broader validation is required, which should involve tests to distinguish between more similar bacteria, such as different species within the same Gram-positive or Gram-negative class, to determine how effectively the measured dielectric contrast can be used to reliably identify different bacteria.

Wang et al.^52^ demonstrated THz near-field imaging of individual bacteria with spatial resolutions of 0.11 to 0.63  μm, whereas our SEM-confirmed tip apex radius of ≈50  nm enables ≈50  nm resolution, an improvement of 2−10×. Lucidi et al.^55^ reported IR-band s-SNOM/AFM topography and amplitude/phase images for 15 bacterial species, but IR imaging poses phototoxicity and thermal damage risks, which THz avoids due to its low photon energy. Although neither study performed full-area n/κ mapping, we provided quantitative n/κ profiles via representative line scans to distinguish Gram-negative and Gram-positive bacteria. These advances underscore the unique capabilities of THz s-SNOM for noninvasive, label-free subsurface biochemical heterogeneity imaging at true nanoscale resolution.

## 4 Conclusion

This study successfully demonstrates the capability of THz s-SNOM to achieve nanoscale imaging and quantitative differentiation of individual bacterial cells based on their intrinsic THz frequency properties. High-resolution imaging (∼50  nm) of individual *E. coli* (Gram-negative) and *B. subtilis* (Gram-positive) bacteria on a gold substrate was realized. A distinct and statistically significant contrast in the THz near-field scattering amplitude was consistently observed, with *E. coli* exhibiting lower signal intensity than *B. subtilis*. Quantitative analysis employing the LDIM further translated this amplitude difference into distinct calculated dielectric functions (or refractive indices) for the two species. We attribute these species-dependent differences to variations in cell envelope structure and hydration. Specifically, the thicker peptidoglycan layer and higher water content of *B. subtilis* result in higher effective permittivity compared with the thinner peptidoglycan layer and complex outer membrane of *E. coli*. We also demonstrated that differences in THz signal between the cell center and edge arise from variations in tip–sample interaction volume and internal hydration distribution. By providing nanoscale spatial resolution and sensitivity to inherent dielectric properties without the need for labeling or destructive preparation, THz s-SNOM emerges as a powerful analytical tool for single-cell scale microbiology. This study highlights its potential for applications requiring high-resolution analysis, such as label-free pathogen identification at the subcellular level, investigating cellular responses to external stimuli such as antimicrobial agents, and advancing fundamental understanding of microorganism biophysics in the THz region. A potential future direction to overcome the strong THz absorption by water is the implementation of an attenuation total reflection configuration, an indirect measurement method common in other spectral ranges. This approach could theoretically allow for probing live cells in their native buffer. However, significant technical challenges, particularly the weak signal strength inherent in probing with an evanescent wave, would need to be addressed to make this feasible. Such advancements could provide deeper insights into the dynamic THz response related to live-cell activity and viability.

## Data Availability

Datasets and analysis codes for this article are available upon request to Prof. Haewook Han, hhan@postech.ac.kr.
